# Considerable effects of lateralization and aging in intracortical excitation and inhibition

**DOI:** 10.3389/fnins.2023.1269474

**Published:** 2023-11-16

**Authors:** Zhongfei Bai, Feifei Zhu, Xiaoyu Lou, Jack Jiaqi Zhang, Minxia Jin, Wenting Qin, Chaozheng Tang, Jie Li, Jiani Lu, Jianhua Lin, Lingjing Jin, Qi Qi, Kenneth N. K. Fong

**Affiliations:** ^1^Department of Rehabilitation, Shanghai YangZhi Rehabilitation Hospital (Shanghai Sunshine Rehabilitation Centre), School of Medicine, Tongji University, Shanghai, China; ^2^Department of Rehabilitation Sciences, The Hong Kong Polytechnic University, Kowloon, Hong Kong SAR, China; ^3^Capacity Building and Continuing Education Center, National Health Commission of the People's Republic of China, Beijing, China; ^4^School of Electronic and Information Engineering, Tongji University, Shanghai, China

**Keywords:** lateralization, aging, TMS-EEG, motor-evoked potentials, intracortical inhibition

## Abstract

**Introduction:**

Findings based on the use of transcranial magnetic stimulation and electromyography (TMS-EMG) to determine the effects of motor lateralization and aging on intracortical excitation and inhibition in the primary motor cortex (M1) are inconsistent in the literature. TMS and electroencephalography (TMS-EEG) measures the excitability of excitatory and inhibitory circuits in the brain cortex without contamination from the spine and muscles. This study aimed to investigate the effects of motor lateralization (dominant and non-dominant hemispheres) and aging (young and older) and their interaction effects on intracortical excitation and inhibition within the M1 in healthy adults, measured using TMS-EMG and TMS-EEG.

**Methods:**

This study included 21 young (mean age = 28.1 ± 3.2 years) and 21 older healthy adults (mean age = 62.8 ± 4.2 years). A battery of TMS-EMG measurements and single-pulse TMS-EEG were recorded for the bilateral M1.

**Results:**

Two-way repeated-measures analysis of variance was used to investigate lateralization and aging and the lateralization-by-aging interaction effect on neurophysiological outcomes. The non-dominant M1 presented a longer cortical silent period and larger amplitudes of P60, N100, and P180. Corticospinal excitability in older participants was significantly reduced, as supported by a larger resting motor threshold and lower motor-evoked potential amplitudes. N100 amplitudes were significantly reduced in older participants, and the N100 and P180 latencies were significantly later than those in young participants. There was no significant lateralization-by-aging interaction effect in any outcome.

**Conclusion:**

Lateralization and aging have independent and significant effects on intracortical excitation and inhibition in healthy adults. The functional decline of excitatory and inhibitory circuits in the M1 is associated with aging.

## Introduction

Lateralization accounts for the variability in motor performance in healthy adults. Using transcranial magnetic stimulation and electromyography (TMS-EMG) outcomes, previous studies have shown that lateralization is associated with asymmetric neurophysiological properties of the motor system (Reid and Serrien, 2012). Additionally, aging gradually deteriorates motor learning and performance as a function of neural degeneration (Voelcker-Rehage, 2008), also resulting in alterations in neurophysiological properties (Ferreri et al., 2017). Previous studies have reported an interaction effect between lateralization and aging on motor function outcomes (Sebastjan et al., 2017), but it seems the two factors have stable effects in the intracortical excitation and inhibition of the motor system over the adult lifespan (Hehl et al., 2022). Understanding the effects of lateralization and aging can provide more evidence related to the deterioration of motor function in elderly people and may be useful for the rehabilitation of geriatric diseases.

The dominant and non-dominant hemispheres are not significantly different in resting motor threshold (RMT) and motor-evoked potentials (MEPs), regardless of handedness (Brouwer et al., 2001; Livingston et al., 2010), suggesting comparable corticospinal excitability between bilateral motor systems at rest. However, inconsistent findings have been reported. For instance, RMT of the non-dominant hemisphere is significantly lower than that of the dominant hemisphere in left-handed young adults (Davidson and Tremblay, 2013); the MEP amplitude of the dominant hemisphere tends to be larger than that of the non-dominant side, particularly in right-handed adults (De Gennaro et al., 2004). Previous studies have extensively investigated the effects of aging on corticospinal excitability and reported the distinct neurophysiological properties of the motor system in young and older adults. The RMT of older adults is slightly higher than or comparable to that of young adults, and their single-pulse induced MEPs are lower (Oliviero et al., 2006; Smith et al., 2009; Young-Bernier et al., 2012; Rozand et al., 2019), suggesting a reduction in corticospinal excitability in older adults. However, it was found that cortical silent period (cSP) was shortened (Oliviero et al., 2006), prolonged (McGinley et al., 2010) or not significantly changed (Fujiyama et al., 2009) in older adults compared with young adults. Similarly, short-interval intracortical inhibition (SICI) is steeper (Smith et al., 2009; McGinley et al., 2010), weaker (Marneweck et al., 2011; Heise et al., 2013), or not significantly changed (Oliviero et al., 2006; Opie and Semmler, 2014), leading to inconclusive aging effects.

Aging-related neural degeneration occurs not only in the brain, but also in the spinal cord, including progressive alpha motor neuron loss which is an essential impairment of neural transmission from the primary motor cortex (M1) to peripheral muscles (Manini et al., 2013; Piekarz et al., 2020). Because of high dependence of motor output in TMS-EMG measures, previous findings with TMS-EMG measures could not purely reveal aging-related alterations in intracortical excitation and inhibition within the M1. The disadvantage of TMS-EMG has been overcome using concurrent TMS and electroencephalography (TMS-EEG), which records the summation of post-synaptic excitatory and inhibitory potentials in response to TMS pulses (Tremblay et al., 2019). The signals evoked by TMS are termed TMS evoked potentials (TEPs), consisting of P30, N45, P55, N100, and P180. Pharmacological TMS studies have revealed that specific TEP peaks are associated with excitatory or inhibitory post-synaptic potentials mediated by NMDA or GABA receptors, respectively. For example, the amplitude of N45 is enhanced by positive modulators of GABA-A receptors, whereas baclofen, a GABA-B receptor agonist, significantly increases the amplitude of N100 (Premoli et al., 2014; Darmani et al., 2016; Gordon et al., 2023). Detailed pharmacophysiology of TEPs has been reviewed by Darmani et al. (2019).

A number of recent studies have recorded TEPs from bilateral M1 stimulation and analyzed interhemispheric inhibition by calculating interhemispheric signal propagation (Casula et al., 2020; Ishibashi et al., 2022). However, only a few studies have investigated hemispheric differences in peak amplitudes of TEPs recorded from dominant and non-dominant M1 simulations. Generally, aging did not significantly alter TEPs recorded from left frontal cortex stimulation (Casarotto et al., 2011), but older adults presented generalized decreased late TEPs recorded from left M1 stimulation, including decreased amplitudes of N44, P60, and N100 (Ferreri et al., 2017), and varied peak latencies (Opie et al., 2018).

In view of previous inconsistent findings and the disadvantage of TMS-EMG measuring in indexing the intracortical excitation and inhibition of the motor system, we aimed to further investigate the effects of lateralization (dominant and non-dominant hemispheres) and aging (young and older), and their interaction effects on intracortical excitation and inhibition within M1. In this study, older adults and young participants were recruited for TMS-EMG measurements, and suprathreshold single-pulse TMS-EEG was performed for bilateral M1. We hypothesized that lateralized intracortical excitability and aging-induced alteration can be indexed by our measurements, and the lateralized intracortical excitability is stable across young and older adults.

## Methods

### Participants

Twenty-one healthy older adults (age: 62.8 ± 4.2 years, 55–68 years; six women) were recruited to participate in this cross-sectional study through poster advertising at the university campus. Additionally, 21 young adults (age: 28.1 ± 3.2 years, 21–33 years; seven women) were recruited from the university. The inclusion criterion was right-handedness according to the Edinburgh Handedness Inventory (Veale, 2014). Individuals were excluded from the study if they had any known history of neurological or psychiatric diseases, were taking neuropsychiatric drugs, or had contraindications to TMS (Rossi et al., 2011). This study was approved by the Human Research Ethics Sub-Committee of The Hong Kong Polytechnic University (Reference Number: HSEARS20200621001) and conducted in accordance with the Declaration of Helsinki. Written informed consent was obtained from all the participants.

### Transcranial magnetic stimulation

All TMS procedures were performed on the dominant and non-dominant hemispheres over an EEG cap placed on the participant’s head. A figure-eight cooling coil (Cooling B-65, external diameter of each wing: 75 mm) connected to a magnetic stimulator (model X100 option, MagVenture A/S, Denmark) was used to deliver the biphasic TMS pulses. To effectively stimulate M1, the coil was placed approximately 45° away from the midline with the handle pointed backward and laterally (Lazzaro et al., 2008). Coil positioning on the scalp was continuously monitored using a frameless stereotactic neuronavigation system (Localite, Bonn, Germany). A motor hotspot was defined as the position at which the largest and most reliable MEPs could be obtained from the first dorsal interosseous muscle contralateral to the stimulation site. Surface EMG activity was recorded using disposable Ag-AgCl electrodes positioned in a belly tendon montage, and a ground electrode was placed on the ulnar styloid process. The raw EMG signals were digitized at 5 kHz, and a Butterworth bandpass filter (fourth-order, 10 Hz–2 kHz) was applied for offline analysis.

A battery of TMS-EMG measurements was performed. The resting motor threshold (RMT) was defined as the minimum intensity (measured as the % of the maximal stimulator output or MSO) that could elicit peak-to-peak MEP amplitudes higher than 50 μV in at least five out of 10 trials. The intensity of the test pulses was set at 120% of the RMT. Single-pulse MEPs were used to measure corticospinal excitability at rest. Short-interval intracortical inhibition (SICI) and intracortical facilitation (ICF) were assessed by delivering a test pulse after a subthreshold conditioning pulse at 80% of the RMT with inter-pulse intervals of 2 and 10 ms, respectively. The ICF and SICI values were calculated as the ratio of MEPs produced by paired-pulse protocols to those produced by single pulses at rest. Cortical silent period (cSP) was the disruption of background EMG activity by a suprathreshold test pulse while sustaining 30% of the maximal voluntary strength of thumb-index finger contraction (Werhahn et al., 1999). The duration of cSP was defined as the time from TMS pulse onset to the first point of a 5-ms window, at which 50% of the EMG signal data samples returned to a level of at least three-fold standard deviations from the silent period. We also quantified MEP amplitudes of active MEPs (aMEPs) in the cSP measurement. Eight trials were recorded for each protocol (inter-trial intervals: 4–5 s) and averaged to obtain the grand means for further statistical analyses.

Concurrent TMS-EEG recordings were performed using a TMS-compatible DC EEG system (SynAmps, NeuroScan) with 64 Ag/AgCl electrodes mounted according to the international 10–10 system. The raw data were referenced online to FCz, grounded to AFz, digitized at a sampling rate of 5 kHz, and filtered online below 2 kHz (Bai et al., 2021). The impedance between the scalp and the electrodes was maintained below 5 kΩ. During recording, 90 TMS pulses at 110% of RMT were applied to M1, with inter-trial intervals of 4–5 s. To suppress the auditory-evoked potentials produced while the coil was discharged, all participants wore an inserted earphone and white noise was played (Tremblay et al., 2019). To minimize the TMS-decay artifacts, a thin piece of foam (3 mm thick) was placed underneath the coil to prevent direct contact with the electrodes (Tremblay et al., 2019), and the direction of the lead wires near the coil was rearranged so that they were perpendicular to the coil (Sekiguchi et al., 2011).

### EEG signal processing

The TMS-EEG signals were pre-processed offline using EEGLAB (Delorme and Makeig, 2004), the TESA extension (Rogasch et al., 2017), FieldTrip (Oostenveld et al., 2011), and custom-made MATLAB scripts. Continuous signals were segmented into individual trials (−2000 to 1999 ms) and baseline-corrected (−500 to −10 ms). Poor trials and channels were excluded from the analysis. The numbers of TEP trials in young (89.6 ± 1.3) and older (87.2 ± 3.1) participants were comparable. The data around the TMS pulses (−2 to 15 ms) were removed and interpolated using a cubic method, followed by a down-sampling procedure to 1 kHz. Thereafter, two rounds of FastICA (systematic approach and tanh contrast function) were performed. The first round aimed to remove the largest TMS-decay artifact detected by a semi-automated component classification algorithm implemented in the TESA. The signals were bandpass-filtered (1–80 Hz) and bandstop-filtered (48–52 Hz) using a fourth-order Butterworth filter, followed by another segmentation from −1,000 to 999 ms. FastICA was conducted again to remove the remaining physiological artifacts. The excluded channels were interpolated back and the reference channel was recovered. Finally, the EEG signals were referenced to a common average and the TEPs were obtained by averaging across trials.

With reference to previous literature (Tremblay et al., 2019), we first predefined five peaks in the temporal domain: P30 (29–35 ms), N45 (42–48 ms), P60 (57–63 ms), N100 (70–110 ms for young participants, 90–130 ms for older participants), and P180 (180–230 ms for young participants, 180–250 ms for older participants). Second, based on the topographical plot of grand-averaged TEPs of all participants, several electrodes were selected to predefine the region of interest for each TEP peak with right stimulation, for example, P30 (C2, C4, CP2, CP4), N45 (AF3, F1, F3, FC1, FC3), P60 (CP4, CP6, P4, P6), N100 (FC1, FCz, FC2, C1, Cz, C2), and P180 (F1, Fz, F2, FC1, FCz, FC2). Third, the amplitude of the TEP peak was obtained by averaging over its predefined time window and region of interest.

### Statistical analysis

Statistical analyses were performed using SPSS22 (IBM, NY, United States). The alpha threshold was set to 0.05 (two-tailed). The peak-to-peak amplitudes of single-pulse MEPs and aMEPs were log-transformed to decrease the inter-participant variability. The normality of variables prior to parametric tests was checked using both one-sample Kolmogorov–Smirnov tests and histogram plots. The TMS-EMG measures and TEP peaks were subjected to two-way repeated measures analysis of variance (rmANOVA) with two main effects (lateralization and aging) and a lateralization-by-aging interaction effect.

## Results

### TMS-EMG measures

**Figure 1:**
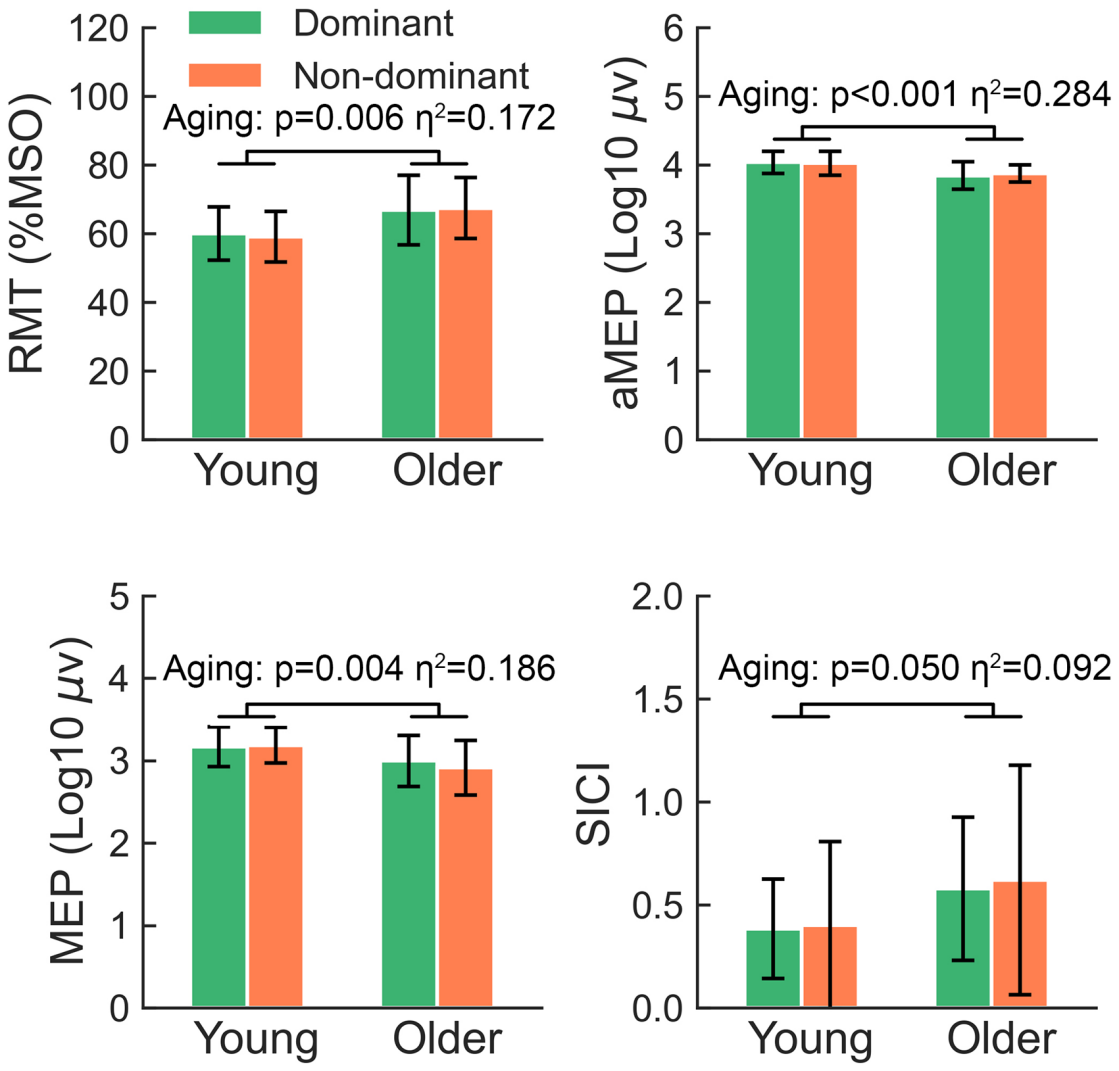
The significant main effects of aging on RMT, aMEP, MEP, and SICI. RMT, resting motor threshold; aMEP, active motor-evoked potential; MEP, motor-evoked potentials; SICI, short-interval intracortical inhibition.

A two-way rmANOVA showed that the RMT of older participants was significantly higher than that of younger participants (*F* = 8.31, *p* = 0.006) (Figure 1). In contrast, older participants showed significantly lower amplitudes of aMEPs (*F* = 15.90, *p* < 0.001) and MEPs (*F* = 9.12, *p* = 0.004) than younger participants. Regarding the comparison of SICI, there was a significant aging effect (*F* = 4.07, *p* = 0.050), suggesting that older participants tended to present with a reduction in GABA-A receptor-mediated intracortical inhibition within M1.

**Figure 2:**
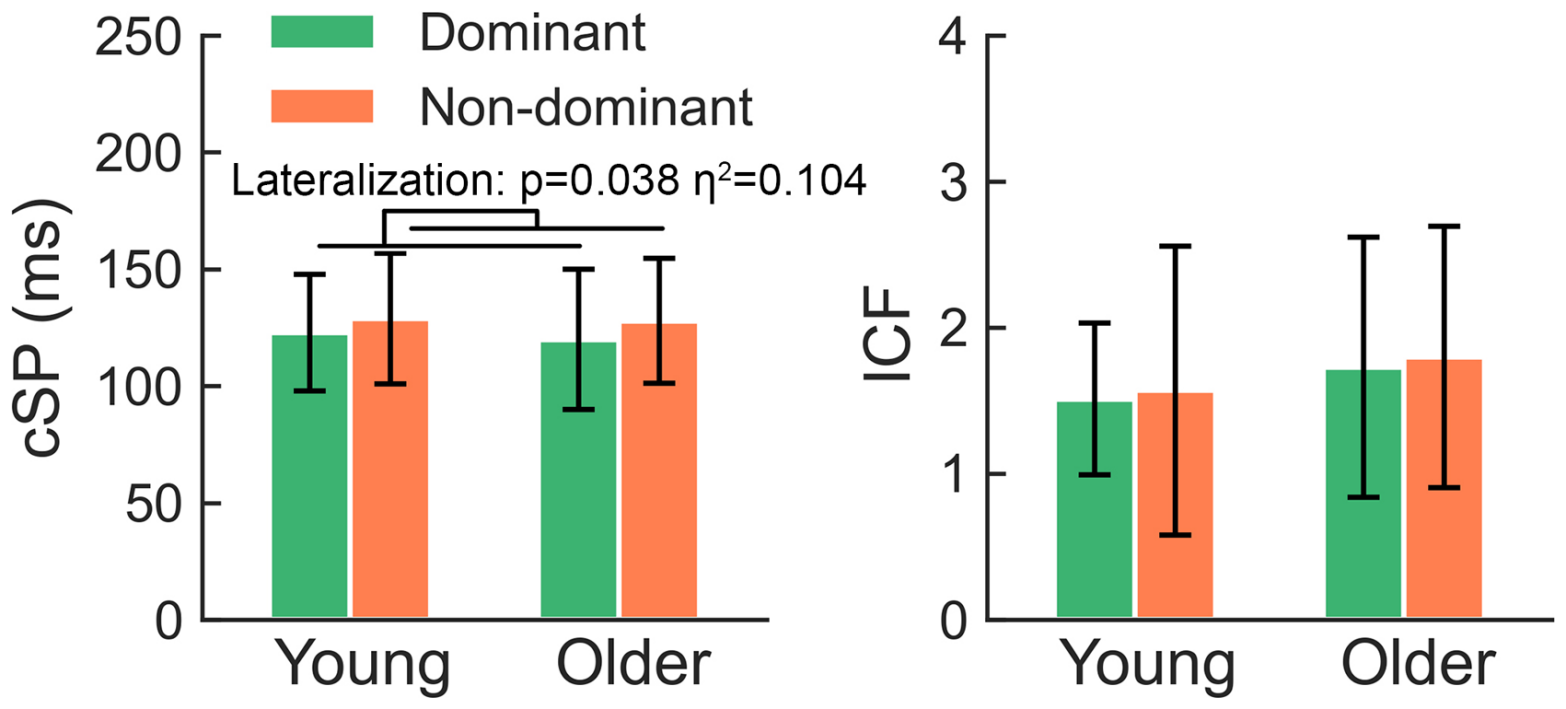
The non-dominant hemisphere presents significantly longer cSP than the dominant side, while comparison of ICF failed to yield any significant effects. cSP, cortical silent period; ICF, intracortical facilitation.

The two-way rmANOVA showed a significant lateralization effect in the comparison of cSP (*F* = 4.63, *p* = 0.038) (Figure 2), suggesting that the cSP of the non-dominant M1 was significantly longer than that of the dominant M1; the aging (*F* = 0.06, *p* = 0.807) and lateralization-by-aging effects (*F* = 0.09, *p* = 0.771) were not significant.

The comparison of ICF failed to yield any significant effects (all *p* > 0.05). There was no significant hemisphere or lateralization-by-aging effect in the comparison of RMT, aMEPs, MEPs, or SICI (all *p* > 0.05).

### TMS-evoked potentials

**Figure 3:**
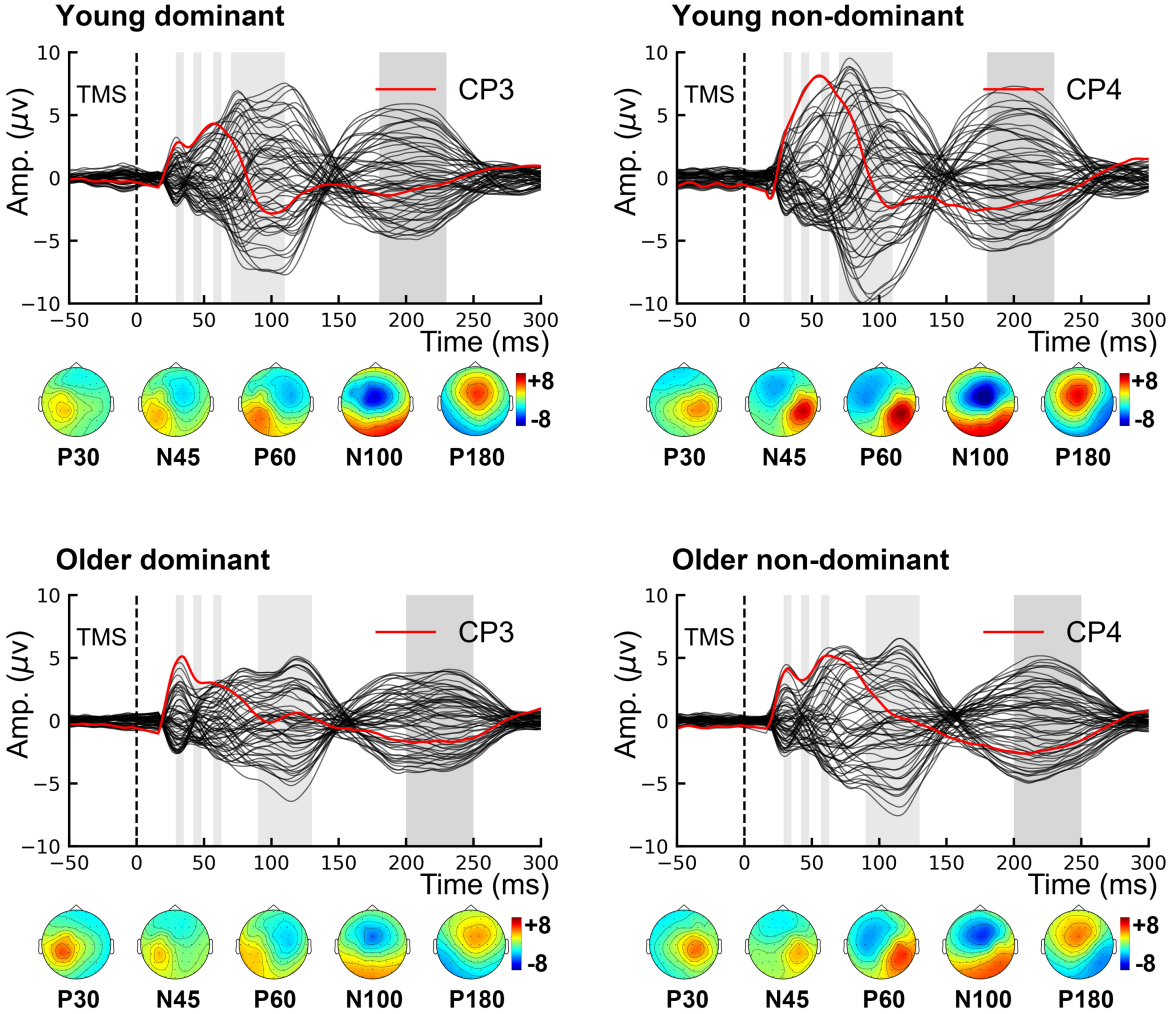
Grand-averaged TMS-evoked potentials. Topographies show the distribution of five TMS-evoked potential peaks. P30 and P60 occur in the stimulated hemisphere, and N45 over the contralateral frontal region. Both N100 and P180 are characterized by bilateral distribution over the centrofrontal region. TMS, transcranial magnetic stimulation.

**Figure 4:**
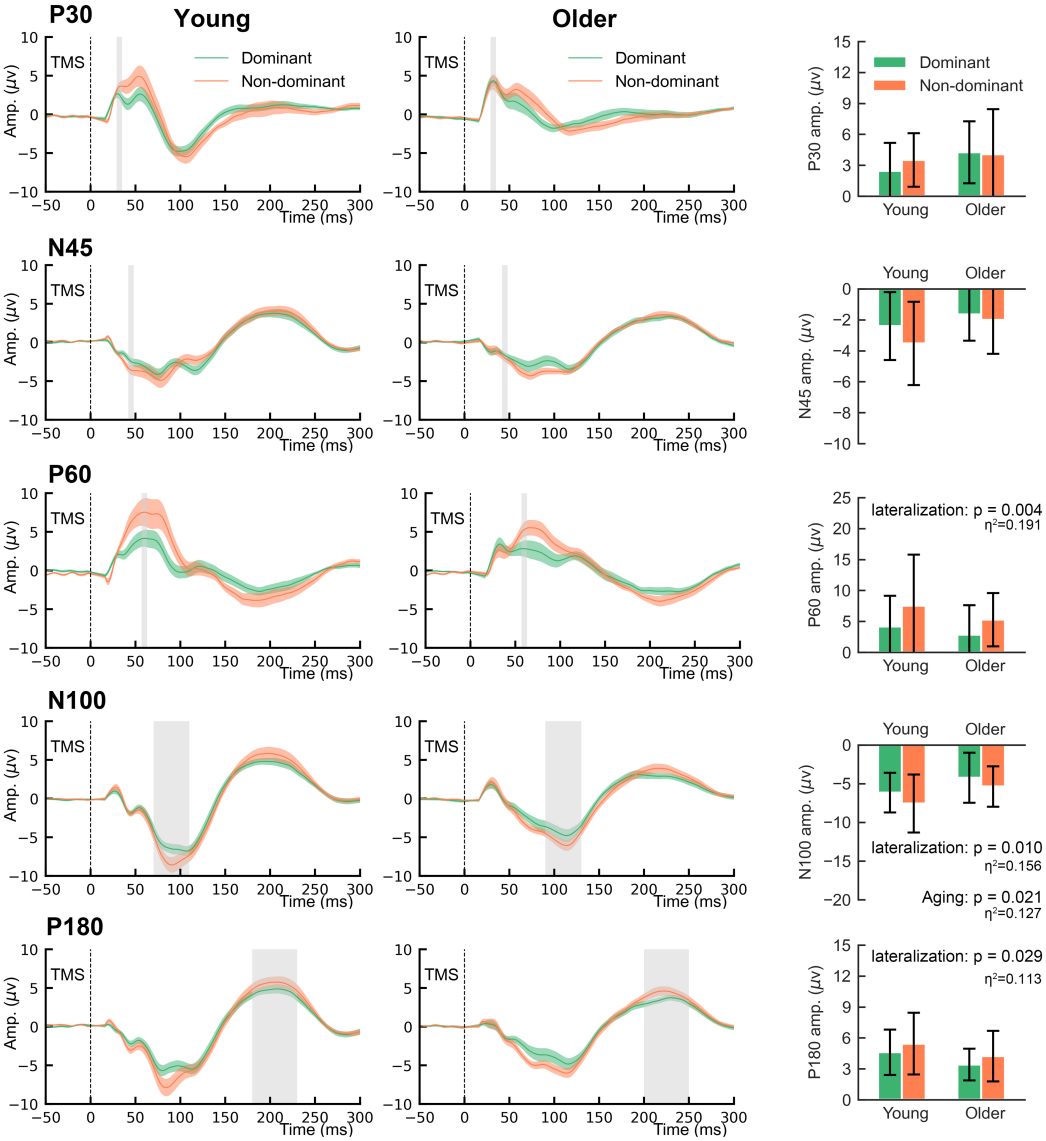
The main effects of lateralization and aging on the amplitudes of TMS-evoked potentials.

The grand-averaged TEP waveforms from bilateral M1 are shown in Figure 3. The comparison of the TEP amplitudes is presented in Figure 4. Two-way rmANOVA failed to show any significant aging (*F* = 1.97, *p* = 0.168) or lateralization (*F* = 0.57, *p* = 0.457) effects on P30. There was no significant lateralization effect on N45 (*F* = 3.82, *p* = 0.058), but a significant lateralization effect on P60 (*F* = 9.41, *p* = 0.004), N100 (*F* = 7.41, *p* = 0.010), and P180 (*F* = 5.10, *p* = 0.029), suggesting that the non-dominant hemisphere had larger amplitudes in these peaks than the dominant hemisphere did.

**Figure 5:**
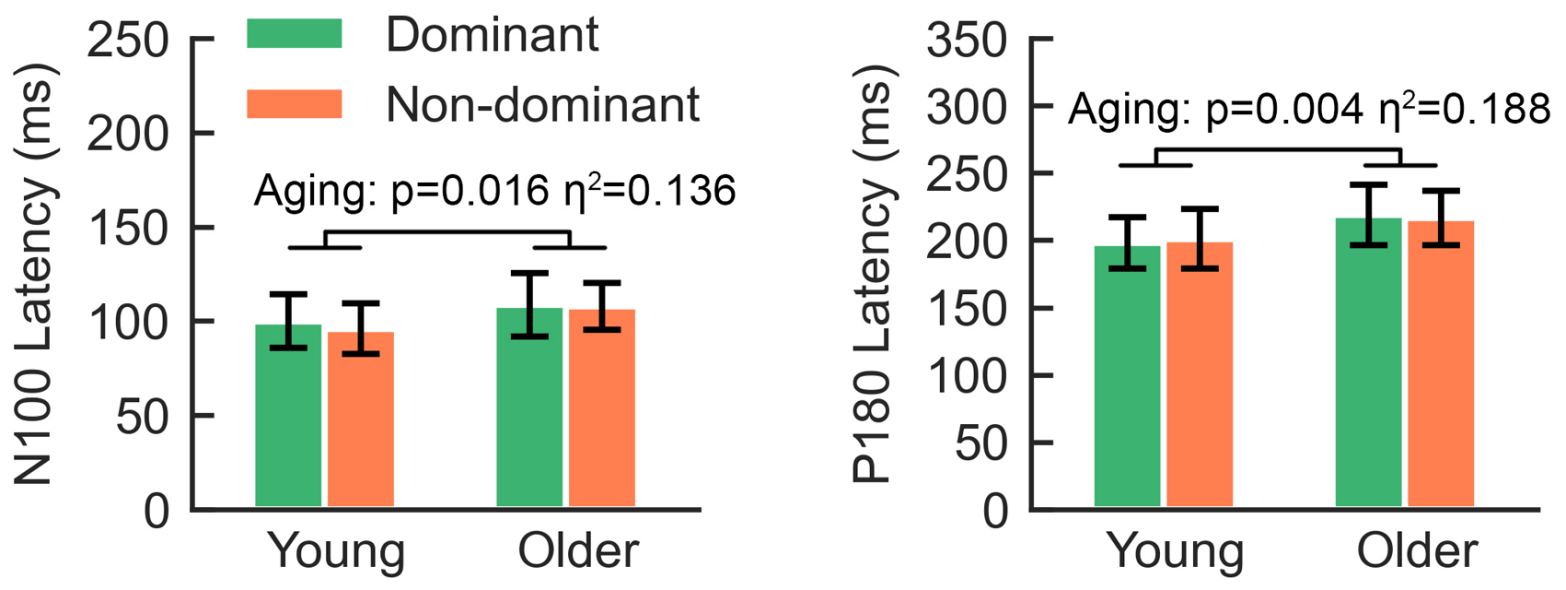
The latencies of N100 and P180 in older adults are significantly longer than those in young adults.

Regarding the aging effect, two-way rmANOVA found statistical significance on N100 (*F* = 5.80, *p* = 0.021), but not on N45 (*F* = 3.66, *p* = 0.063), P60 (*F* = 1.29, *p* = 0.263) or P180 (*F* = 3.44, *p* = 0.071). These findings suggest that older participants tend to present smaller amplitudes on N100. There was no significant lateralization-by-aging effect in any comparison of the five TEPs (all *p* > 0.05). As shown in Figure 3, N100 and P180 peaked at later latencies in older participants than in younger participants. The two-way rmANOVA showed a significant aging effect on the latencies of N100 (*F* = 6.28, *p* = 0.016) and P180 (*F* = 9.25, *p* = 0.004), suggesting that both peaks of older participants occurred significantly later than those of younger participants (Figure 5). There were no significant hemisphere or lateralization-by-aging effects (all *p* > 0.05).

## Discussion

By using TMS-EMG and TMS-EEG, our study showed that lateralization and aging had independent effects in intracortical excitation and inhibition within the M1. Specifically, the non-dominant M1 presented longer cSP and larger amplitudes of P60, N100, and P180. Regarding the effects of aging, the corticospinal excitability of older participants was significantly reduced, supported by larger RMT, lower MEPs, and aMEPs. Simultaneously, significantly reduced amplitudes of N100 were found in older participants, and the latencies of both N100 and P180 were significantly later than those in young participants. Because of non-significant interactions effects, the lateralization of intracortical excitation and inhibition is stable along with aging, reinforcing previous findings (Hehl et al., 2022).

### Lateralization

On the one hand, we found that the dominant and non-dominant M1 was not significantly different in RMT, aMEPs, MEPs, or SICI, in line with previous studies (Brouwer et al., 2001; Livingston et al., 2010). On the other hand, our study replicated the previous finding that the cSP of the non-dominant M1 was significantly longer than that of the dominant M1, which is also in line with previous studies (Priori et al., 1999; Reid and Serrien, 2012). The initial inhibition of cSP occurs at the level of spinal cord, while the later one is due to GABA-B receptor-mediated intracortical inhibition (Chen et al., 1999). Therefore, researchers have proposed that asymmetry of cSP might occur in the spinal cord or cortex (Priori et al., 1999). TMS-EEG can be used to probe cortical responses to TMS pulses without contamination from spinal cord activity, and the N100 amplitude is associated with GABA-B receptor-mediated intracortical inhibition (Premoli et al., 2014). In the present study, the N100 amplitude derived from non-dominant M1 stimulation was significantly larger than that derived from dominant M1 stimulation, suggesting that the difference in bilateral cSP likely occurs in the cortex rather than in the spinal cord.

To date, few studies have investigated hemispheric differences in peak amplitudes of TEPs. In the present study, significantly larger amplitudes of P60 and P180 were observed in the non-dominant M1 stimulation, but the mechanisms of P60 and P180 are not clearly enucleated. A recent pharmacological study demonstrated that AMPA receptor antagonists could significantly reduce the amplitude of P60 over the non-stimulated hemisphere, suggesting that AMPA receptors are involved in interhemispheric signal propagation (Belardinelli et al., 2021). However, the larger P60 amplitudes found in the current study were over the stimulated hemisphere, which meant that AMPA receptor-mediated glutamatergic excitation may not be the source of the hemispheric difference. GABAergic drugs were found to have no modulatory effects on the amplitude of P180 (Premoli et al., 2014), but a single oral dose of carbamazepine (a voltage-gated sodium channel blocker) decreased the amplitude of P180 (Darmani et al., 2019). Although these hemispheric differences have been confirmed, their implications in terms of motor performance and/or learning are not clear and further studies are needed.

### Aging

In line with previous studies using TMS-EMG, we observed reduced corticospinal excitability in older participants (Brouwer et al., 2001; Livingston et al., 2010). Regarding the effect of aging on intracortical inhibition, largely inconsistent findings have been reported, and no consensus has been reached. This discrepancy may be caused by methodological variations among studies (Opie and Semmler, 2014), which are vital for the inhibitory effect of SICI. For instance, the greatest SICI is yielded by a test pulse at an intensity of 110–120% RMT, and higher test intensities reduce the inhibition of SICI (Garry and Thomson, 2008). The intensity of a conditional pulse and inter-pulse interval also have interaction effects on SICI (Vucic et al., 2009). In the present study, older participants presented with reduced SICI. Simultaneously, we also found that the N45 amplitude of older participants tended to be smaller than that of younger participants, in line with a previous study (Noda et al., 2021), providing further evidence that aging reduces GABA-A receptor-mediated intracortical inhibition, which can contribute to the deterioration of motor function (Levin et al., 2014).

The N100 amplitude and duration of cSP are believed to be mediated by GABA-B receptors (Stetkarova and Kofler, 2013; Premoli et al., 2014). However, dissociation of the two measures was found in the current study. Specifically, the N100 amplitude decreased in older participants, but the duration of cSP was not significantly changed by aging, suggesting that N100 and cSP represent the magnitude and duration of GABA-B receptor-mediated intracortical inhibition, respectively. N100 has not been extensively investigated in older adults; however, many previous studies have shown that long-interval intracortical inhibition, having a similar mechanism to that of N100 (Premoli et al., 2014; Darmani et al., 2016), is reduced in older adults (Opie and Semmler, 2014; Hermans et al., 2018, 2019). Therefore, these multiple-model data support the notion that the magnitude of GABA-B receptor-mediated intracortical inhibition is reduced in older adults, which may be a compensatory adaptation for reduced corticospinal excitability (Kallioniemi et al., 2022). A contradictory aging effect on the duration of cSP has been reported in the literature, and we found a non-significant aging effect on it. One possible explanation for this discrepancy is the different intensities used, which influence the duration of the cSP (Säisänen et al., 2008). Further studies should employ multiple levels of suprathreshold intensity to comprehensively test how aging modulates cSP. In addition to the significantly modulated TEP amplitudes due to aging, our analysis further demonstrated later latencies of N100 and P180, which is partially consistent with a previous study (Opie et al., 2018). The mechanism of TEP latency has not been fully elucidated and is probably associated with nerve conduction velocity and the reactivity of specific neural transmitters to TMS pulses (Noda et al., 2021). Further studies should investigate how these neurophysiological biomarkers are related to behavioral changes in older adults.

This study had some limitations. First, all participants were right-hand dominant, but appropriate measures were not carried out to evaluate the behavioral asymmetry of the bilateral hands, which may weaken the implications of the current study. Second, the average age of the older participants was lower than that in previous studies, decreasing the comparability between the current study and others. Finally, our analysis was conducted for predefined ROIs, in which the largest amplitude of a certain peak was. Whether there were hemispheric and/or aging-related differences in TEPs beyond predefined ROIs is not clear. Fourth, white nose masking was used to suppress auditory-evoked potentials during TMS-EEG recording, but a sham control condition was not employed to rule out the contamination of somatosensory-evoked potentials.

In conclusion, our study found that lateralization and aging have significant effects on intracortical excitation and inhibition, and such lateralization effects are stable across young and older adults. Longer cSP and larger amplitudes of P60, N100, and P180 were observed in the non-dominant M1. Older participants presented lower corticospinal excitability and N100 than young participants, suggesting that the function of excitatory and inhibitory circuits in M1 might be reduced due to aging. The latencies of both N100 and P180 were later than those of young participants, which requires further studies on how these neurophysiological biomarkers are associated with behavioral changes during normal aging.

## Data availability statement

The raw data supporting the conclusions of this article will be made available by the authors, without undue reservation.

## Ethics statement

This study was approved by the Human Research Ethics Sub-Committee of The Hong Kong Polytechnic University (Reference Number: HSEARS20200621001). The studies were conducted in accordance with the local legislation and institutional requirements. The participants provided their written informed consent to participate in this study.

## Author contributions

ZB: Data curation, Formal analysis, Funding acquisition, Investigation, Methodology, Project administration, Visualization, Writing – original draft. FZ: Data curation, Investigation, Visualization, Writing – original draft. XL: Data curation, Visualization, Writing – original draft. JZ: Data curation, Formal analysis, Writing – original draft. MJ: Investigation, Writing – original draft. WQ: Writing – original draft. CT: Writing – original draft. JLi: Writing – original draft. JLu: Writing – review & editing. JLin: Writing – review & editing. LJ: Formal analysis, Investigation, Writing – review & editing. QQ: Conceptualization, Project administration, Resources, Writing – review & editing. KF: Conceptualization, Data curation, Project administration, Resources, Supervision, Writing – review & editing.
